# Verified Certification of Reachability Checking for Timed Automata

**DOI:** 10.1007/978-3-030-45190-5_24

**Published:** 2020-03-13

**Authors:** Simon Wimmer, Joshua von Mutius

**Affiliations:** 8 Johannes Kepler University, Linz, Austria; 9 University of Birmingham, Birmingham, UK; Fakultät für Informatik, Technische Universität München, Munich, Germany

**Keywords:** Timed automata, Certification, Model Checking, Interactive Theorem Proving, Isabelle/HOL

## Abstract

Prior research has shown how to construct a mechanically verified model checker for timed automata, a popular formalism for modeling real-time systems.

In this paper, we shift the focus from verified model checking to certifying unreachability. This allows us to benefit from better approximation operations for symbolic states, and reduces execution time by exploring fewer states and by exploiting parallelism. Moreover, this gives us the ability to audit results of unverified model checkers that implement a range of further optimizations, including certificate compression.

The resulting tool is evaluated on a set of standard benchmarks to demonstrate its practicality, using a new unverified model checker implementation in Standard ML to construct the certificates.

Timed automata [1] are a widely used formalism for modeling real-time systems, which is employed in a class of successful model checkers such as UPPAAL [4]. These tools can be understood as trust-multipliers: we trust their correctness to deduce trust in the safety of systems checked by these tools. As a consequence, one wants to ensure as rigorously as possible that the computation results of timed automata model checkers are correct.

Previous work [31] has addressed this problem by constructing a model checker for timed automata that is fully verified using Isabelle/HOL [25]. This tool is intended to be a reference implementation that can be used to scrutinize the correctness of other model checkers. As such, it is mainly able to check small and medium-sized benchmark examples, but the performance gap w.r.t. more practical model checkers prevents it from checking realistic benchmark models within reasonable time and space bounds.

We address this issue by shifting the focus from full verified model checking to only certifying that the result produced by an unverified model checker is correct. We only study reachability: it is the most important property that is checked with timed automata model checkers, and some model checkers only support reachability. It is crucial to ensure that a bad state is certainly not reachable if the model checker claims so, thus we want to certify *unreachability*. Certifying that a state is indeed reachable would amount to extracting a timed trace and certifying that the trace is compatible with the model. While implementing this in a verified manner would be comparatively easy, we consider it less important because it corresponds to the bug finding functionality of model checkers, which carries less trust.

The recipe for certifying unreachability is simple: the model checker explores a number of states until it determines that there are no more states to be found. If none of the states fulfill the final state predicate (i.e. violates the safety property), then the model checker will answer "unreachable". We use the set of explored states as the unreachability certificate. In essence, we only need to check that the initial state is contained in this set, that there are no outgoing edges from this set, and that none of the states in the set fulfill the final state predicate.

The switch to certification holds many advantages. Timed automata model checking uses *over-approximations* of symbolic states to ensure termination. A large variety of these approximation operators has been studied [2,3,14]. Our previous work [29] has shown that, while formally proving the correctness of these approximation operations is feasible in principle with an interactive theorem prover, the effort is rather high. Instead, to *certify* unreachability, it is sufficient to only know that the approximation operator indeed yields a state that is at least as big as the precise symbolic state. Certifying this property is cheap.

Moreover, certification eases *parallelization*. Checking that a state is not final and that all its successors are covered by the state set are local properties. We show how to exploit this in a verified implementation, while only mildly increasing the verification effort and the size of the trusted code base.

Finally, the number of states explored by a model checker can vary immensely, depending on a range of factors such as the chosen approximation operator or the search order. Thus, an efficient unverified tool can exploit different heuristics and strategies to compute a state space that is as small as possible, and thereby speedup the certification effort. In this context, we also study a number of *compression techniques* to reduce the number of states in the certificate after the model checker has concluded its search.

We use a new unverified model checker called Mlunta, which is implemented in Standard ML (SML), to generate certificates for a set of standard benchmarks, and to evaluate our verified certifier's performance on these benchmarks [1].

*Related Work* This work is based on an existing Isabelle/HOL formalization of timed automata model checking [29,31]. Other proof-assistant formalizations of timed automata focus on proving elementary properties about the basic formalism [33,34], or proving properties about concrete automata [26,10,8], but none of them are concerned with model checking.

Earlier work formalizes a model checker for the modal $\mu$-calculus [28], and constructs a verified finite state LTL model checker [9,24,6].

[1] Both tools are available online: https://doi.org/10.5281/zenodo.3679245.

The idea of extracting certificates from the model checking process has previously been studied in the context of the $\mu$-calculus [23] and finite state LTL model checking [27]. However, these works are not accompanied by a verified certificate checker and do not attempt to scale the approach to practical examples. Only the recent work of Griggio et al. [11] provides a practical extraction mechanism and a certificate checker for LTL model checking, but the checker is not verified. To the best of our knowledge, we are the first to examine certification in the context of timed automata model checking.

Finally, in the context of software verification, the idea of producing certificates for the correctness of a program has been broadly studied [16,5].

*Isabelle/HOL* Isabelle/HOL [25] is an interactive theorem prover based on Higher-Order Logic (HOL). HOL can be thought of as a combination of a functional programming language and mathematical logic. Isabelle/HOL mostly resembles standard mathematical notation. Some conventions that are borrowed from functional programming need to be explained, however. Functions are mostly curried, i.e. of type $\tau_1 \Rightarrow \tau_2 \Rightarrow \tau$ instead of $\tau_1 \times \tau_2 \Rightarrow \tau$. As a consequence, function application is usually denoted as $f\ a\ b$ instead of $f(a, b)$. Function abstraction with lambda terms uses the standard syntax $\lambda x.\ t$ (the function that maps $x$ to $t$) and can also have paired arguments $\lambda(x, y).\ t$. Type variables are written $'a$, $'b$, etc. Compound types are written in postfix syntax: $\tau\ set$ is the type of sets of elements of type $\tau$. We use the Isabelle/HOL convention that free variables are implicitly all-quantified throughout the paper. In parts of the paper, formulas or syntax have been simplified for readability, but we have stayed largely faithful to the Isabelle/HOL formalization.

*Contributions* In short, these are the main contributions of our work:

- To the best of our knowledge, we are the first to study certification of the model checking results of reachability checking for timed automata, including techniques to compress certificates.
- We construct a verified implementation of such a certificate checker, including a number of optimization techniques to make it practically usable.

*Outline* The remainder of the paper is organized as follows. The first section briefly recalls the theory of timed automata, and sketches the state-of-the-art model checking process. The second section details our approach to certification and explains how, starting from an abstract theory, a concrete verified implementation of the certificate checker can be obtained. Section three illustrates a number of techniques to improve the certificate checker's performance, while only mildly increasing the formalization effort. Section four discusses two methods for certificate compression. The paper is concluded by an experimental evaluation and remarks on potential future work.

## 1 Timed Automata and Model Checking

*Transition Systems* We take a very simple view of transition systems: they are simply a relation $\rightarrow$ of type $'a \Rightarrow 'a \Rightarrow bool$ for a type of states $'a$. We write $a \rightarrow^* b$ to denote that $b$ can be reached from $a$ via a sequence of $\rightarrow$-transitions.

*Timed Automata* To make the paper self-contained, this paragraph briefly describes timed automata and is mostly reproduced from Wimmer and Lammich [29]. For a thorough introduction see the tutorial paper of Bengtsson and Yi [4].

Compared to standard finite automata, timed automata introduce a notion of clocks. Figure 1 depicts an example of a timed automaton. We will assume that clocks are of type *nat*. A *clock valuation* $u$ is a function of type $nat \Rightarrow real$. Locations and transitions are guarded by *clock constraints*, which have to be

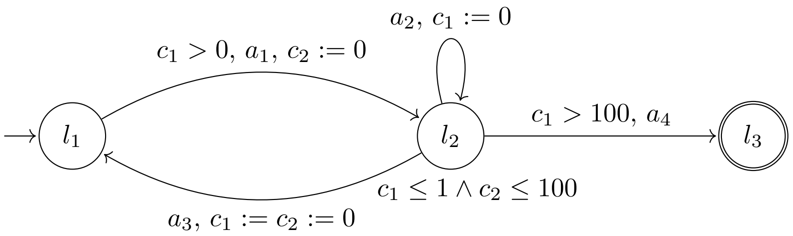

Fig. 1: Example of a timed automaton with two clocks.

fulfilled to stay in a location or to take a transition. Clock constraints are conjunctions of constraints of the form $c \sim d$ for a clock $c$, an integer $d$, and $\sim\, \in \{<, \le, =, \ge, >\}$. We write $u \models cc$ if the clock constraint $cc$ holds for the clock valuation $u$. We define a timed automaton $A$ as a pair $(\mathcal{T}, \mathcal{I})$ where $\mathcal{I}$ is a mapping from locations to clock constraints (also named invariants); and $\mathcal{T}$ is a set of transitions written as $A \vdash l \longrightarrow^{g,a,r} l'$ where $l$ and $l'$ are start and successor location, $g$ is the guard of the transition, $a$ is an action label, and $r$ is a list of clocks that will be reset to zero when the transition is taken. States of timed automata are pairs of a location and a clock valuation. The operational semantics defines two kinds of steps (given as their HOL descriptions):

- Delay: $(l, u) \rightarrow^d (l, u \oplus d)$ if $d \ge 0$ and $u \oplus d \models \mathcal{I}\ l$;
- Action: $(l, u) \rightarrow_a (l', [r := 0]u)$
  if $A \vdash l \longrightarrow^{g,a,r} l'$, $u \models g$, and $[r := 0]u \models \mathcal{I}\ l'$; where $u \oplus d = (\lambda c.\ u\ c + d)$ offsets all clocks by $d$ in the valuation $u$, and $[r := 0]u = (\lambda c.\ \mathsf{if}\ c \in r\ \mathsf{then}\ 0\ \mathsf{else}\ u\ c)$ resets all clocks in $r$ to 0 in valuation $u$. For any (timed) automaton $A$, we consider the transition system

$$(l, u) \rightarrow_A (l', u') = (\exists d \ge 0.\ \exists a\ u''.\ (l, u) \rightarrow^d (l, u'') \land (l, u'') \rightarrow_a (l', u'))\,.$$

That is, each transition consists of a delay step that advances all clocks by some amount of time, followed by an action step that takes a transition and resets the clocks annotated to the transition. Given a final state predicate $F$ and an initial state $(l_0, u_0)$, we are interested in whether $(l_0, u_0) \to_A^* (l, u)$ for any $l$, $u$ with $F\,l$. In Figure 1, the final state is $l_3$ (i.e. $F\,l \longleftrightarrow l = l_3$). As the guard for action $a_4$ is never enabled, $l_3$ is unreachable.

*Model Checking* Due to the use of clock valuations, the state space of timed automata is inherently infinite. Thus, model checking algorithms for timed automata are based on the idea of abstracting from concrete valuations to *sets* of clock valuations of type $(nat \Rightarrow real)\ set$, often called *zones*. The resulting transition system of reachable states from an initial zone is called the zone graph. It is explored in an *on-the-fly* manner, computing successors on zones, which are typically represented symbolically as *Difference Bound Matrices* (DBMs). Knowledge of this data structure is not necessary to understand the rest of the paper. Thus we refer the interested reader to Bengtsson and Yi [4] and to Wimmer and Lammich [29,31] for a verification of this data structure. In the remainder we will only use the term "zones" instead of referring to their implementation as DBMs.

The delicate part of this method is that the number of reachable zones could still be infinite. Therefore, over-approximations (or *abstractions*) of zones are computed to obtain a finite search space. For our purpose, it sufficient to assume an abstraction operator $\alpha$ indeed computes an over-approximation, i.e. $Z \subseteq \alpha(Z)$ for any zone $Z$. We call the version of the zone graph where abstractions are applied the *abstract zone graph* [13]. For a number of such abstraction operators, it can be shown that the abstract zone graph is sound and complete [2]. The proofs are rather intricate, however. Thus formalizing them would be a big effort. By focusing on certification of unreachability, this problem vanishes, as we only need to ensure that any state $(l, Z)$ that we deem reachable in the zone graph is *subsumed* by some state $(l, Z')$ with $Z \subseteq Z'$ that is part of the certificate and that was computed by the abstraction (i.e. $Z' = \alpha(Z_1)$ for some $Z_1$).

*Certificates by Example* Figure 2 depicts the zone graph of the automaton in Figure 1. Each zone $Z$ is given as a clock constraint $cc$ such that $Z = \{u \mid u \models cc\}$. A model checker like Munta would have to explore the full zone graph before being able to decide that $l_3$ is unreachable. Any model checker that uses the same abstraction technique as Munta [2] would not be able to benefit from abstractions for this example and thus the abstract zone graph is the same as the zone graph. However, such a model checker could apply subsumptions while exploring the zone graph. That is, when a symbolic state of the form $(l_2, \{u \mid u \models c_1 = 0 \wedge c_2 < k+1\})$ is explored, the state $(l_2, \{u \mid u \models c_1 = 0 \wedge c_2 < k\})$ can safely be discarded.

This means that at the end of the model checking process, only the three states in Figure 3a will be stored. The solid edges are part of the zone graph,

[2] Soundness: for every abstract run, there is a concrete instantiation. Completeness: every concrete run can be abstracted.

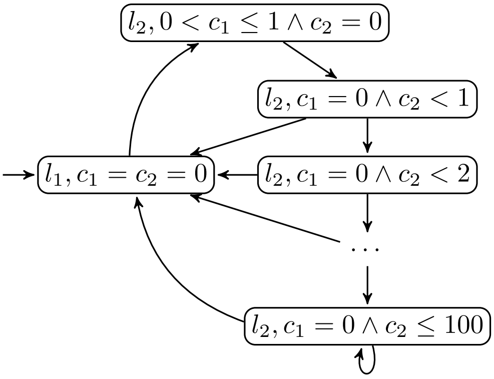

Fig. 2: The zone graph of the automaton depicted in Figure 1.

while the dashed edge indicates that the zone at its tail has a successor in the zone graph $((l_2, \{u \mid u \models c_1 = 0 \wedge c_2 < 1\}))$ that is subsumed by the tip of the edge. The set of these three states can act as a *certificate* of unreachability. They essentially form an inductive invariant of the zone graph: for each state in the certificate, all its successors in the zone graph are either contained in the certificate themselves or subsumed by another state in the certificate. Thus we know that any symbolic state that is reachable from the initial state is subsumed by some state in the certificate, and as the final state is not contained in the certificate, we can conclude that it is unreachable.

Figure 3b shows a certificate with only two states that replaces the two states for $l_2$ by the state with a dashed border. Note that this state is not part of the original zone graph. The certificate fulfills the same invariant property and thus also proves unreachability. We will use this technique of adding larger states to the certificate that are not part of the zone graph for our compression techniques in section 4.

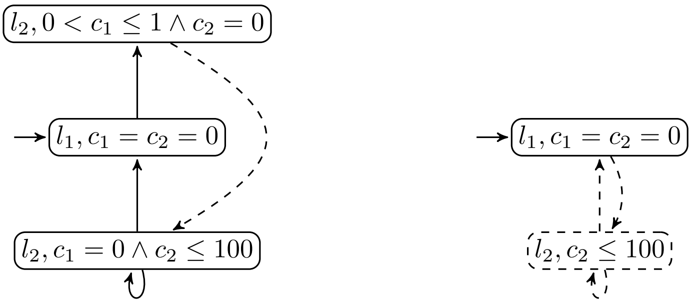

(a) Stored states (b) Smaller certificate

Fig. 3: Two certificates of unreachability for the automaton from Figure 1.

## 2 From Model Checking to Certifying Unreachability

This section first describes our approach to certification abstractly. Then, we detail how the existing formalization of a timed automata model checker was extended—with rather low effort—to a verified certifier. In practice, networks of timed automata with additional modeling features such as, e.g. shared state variables, are used. However, due to the existing verified product construction for such a formalism [31], it is sufficient to study the case of a single timed automaton here.

### 2.1 An Abstract Correctness Theorem

To work towards a rigorous justification of the certification process, we first study the problem on a more abstract level. Consider a transition system $\rightarrow$ on states of type $'l \times 's$ where $'l$ corresponds to the finite state part of timed automata and $'s$ corresponds to zones. We assume an invariant $P$ on states, i.e.:

$$P\ (l_1,\ s_1)\ \wedge (l_1,\ s_1) \rightarrow (l_2,\ s_2) \Longrightarrow P\ (l_2,\ s_2)\,.$$

This invariant essentially represents a restriction of $\rightarrow$ to valid states. While this would usually be assumed implicitly, we explicate $P$ here as it is technically more convenient to do so in the Isabelle/HOL formalization.

The interesting feature that sets timed automata model checking apart is subsumption. Recall that during the model checking process, it is possible to first discover some (symbolic) state $(l, Z)$ (a pair of a discrete state $l$ and a zone $Z$), and to find at some later point that another reachable state $(l, Z')$ subsumes $(l, Z)$ because $Z'$ semantically contains $Z'$, i.e. $Z \subseteq Z'$. At this point the state $(l, Z)$ can be discarded as we know that anything that is reachable from $(l, Z)$ is also reachable from $(l, Z')$. Abstractly, subsumption is modeled by some fixed preorder (i.e. a reflexive and transitive relation) $\preccurlyeq$ on $'s$ which is a simulation relation between $\rightarrow$ and itself:

$$\begin{array}{ll} & s_1 \preccurlyeq s_2 \wedge (l_1, s_1) \rightarrow (l_2, t_1) \wedge P\ (l_1, s_1) \wedge P\ (l_2, s_2) \\ \Longrightarrow & \exists t_2.\ t_1 \preccurlyeq t_2 \wedge (l_1, s_2) \rightarrow (l_2, t_2) \end{array}$$

In the abstract setting, a certificate consists of a set of discrete states $L$ of type $'l\,set$, and a mapping $M$ of type $'l \Rightarrow 's\,set$ that gives the set of reachable symbolic states that were computed for any discrete state $l \in L$. We say that $(L, M)$ satisfies $P$ if all states in the certificate $(L, M)$ satisfy $P$:

$$l\ \in\ L\ \wedge\ s\ \in\ M\,l \Longrightarrow P\,(l,\, s)$$

Moreover, the certificate needs to be *closed*. Following Herbreteau et al. [13], we call a state *covered* if it is subsumed by another state in the certificate. A certificate is closed if for each state in the certificate all its successors are covered:

$$l_1 \in L \wedge s_1 \in M\,l_1 \wedge (l_1,\ s_1) \rightarrow (l_2,\ s_2) \Longrightarrow l_2 \in L \wedge (\exists s_3 \in M\,l_2.\ s_2 \preccurlyeq s_3) \quad (*)$$

The following key theorem states that all reachable states are covered if the initial state is covered:

**Theorem 1.** *Let $(L, M)$ be closed and invariant under $P$. Assume $l_0 \in L$, $s_0' \in M\, l_0$, $s_0 \preccurlyeq s_0'$, and $(l_0,\, s_0) \rightarrow^* (l,\, s)$. Then $l \in L$ and there exists $s'$ such that $s' \in M\, l$ and $s \preccurlyeq s'$.*

*Proof.* By induction on the number of steps in $(l_0,\, s_0) \rightarrow^* (l,\, s)$. The following sketches how the run of covering states is constructed. The first line represents $(l_0, s_0) \rightarrow^* (l, s)$ and the states in the third line are all part of the certificate.

$$
\begin{array}{ccccccc}
(l_0, s_0) & \rightarrow & (l_1, s_1) & \rightarrow & \dots & \rightarrow & (l, s) \\
 & & \preceq & & & & \preceq \\
 & & (l_1, t_1) & & \dots & & (l, t) \\
\preceq & \nearrow & \preceq & \nearrow & & \nearrow & \preceq \\
(l_0, s_0') & & (l_1, s_1') & & \dots & & (l, s')
\end{array}
$$

From the assumptions on $l_0$, $s_0$, and $s_0'$, we can first apply the self-simulation property of $\rightarrow$ to $(l_0, s_0) \rightarrow (l_1, s_1)$ to obtain a $t_1$ such that $s_1 \preccurlyeq t_1$ and $(l_0, s_0') \rightarrow (l_1, t_1)$. As the certificate is closed we thus get $l_1 \in L$ and we can find an $s_1' \in M\, l_1$ such that $t_1 \preccurlyeq s_1'$ (and thus $s_1 \preccurlyeq s_1'$ by transitivity). The induction hypothesis can then be applied to $l_1$, $s_1$, and $s_1'$. □

We will now say that a certificate $(L, M)$ is *admissible* iff

- it satisfies $P$,
- it is closed,
- it covers the initial state (i.e. there is an $s_0' \in M\, l_0$ such that $s_0 \preccurlyeq s_0'$),
- and there is no $l \in L$ with $F\, l$.

**Corollary 1.** *If $F$ is monotone w.r.t. $\preccurlyeq$ and the certificate $(L, M)$ is admissible, then $\nexists l\, s.\, (l_0,\, s_0) \rightarrow^* (l,\, s) \wedge F\, l$.*

#### 2.2 An Abstract Certificate Checker

In practice, the certification process has to consider one additional complication. A model is typically described in terms of human-readable identifiers, while most model checkers and the verified model checker Munta [30] in particular represent these as natural numbers internally to allow for efficient indexing. In our certifier, this is accounted for by relabeling the human-readable identifiers in a given model to natural numbers in a first (verified) pre-processing step. To save additional transformations of the certificate after it was emitted, we let the unverified model checker additionally emit a textual description of such a renaming. The certifier then just needs to check that the given renaming is injective to ensure that it can safely be applied.

Together with the theoretical analysis laid out in the last section, we can thus derive the following strategy for certifying unreachability:

- An unverified model checker explores the reachable state space of a given model symbolically and checks that none of the discovered states $(l, s)$ fulfills $F\, l$.

```
1  definition check (L, M) ≡
2    monadic_list_all L (λl. do {
3      let S = M l;
4      let next = succs l S;
5      monadic_list_all next (λ(l', S'). do {
6        xs ← SPEC (λxs. set xs = S');
7        if xs = [] then return True else do {
8          b1 ← return (l' ∈ L);
9          ys ← SPEC (λxs. set xs = M l');
10         b2 ← monadic_list_all xs (λx.
11           monadic_list_ex ys (λy. return (x ≼ y))
12         );
13         return (b1 ∧ b2)
14       }
15     })
16   })
```

Listing 1.1: Monadic program to check whether a certificate is closed.

- The set of explored states is emitted as a certificate, possibly followed by compression (see section 4).
- The model, the final state predicate $F$, the certificate, and a description of the renaming that was used for the states are passed to the verified certifier.
- The certifier checks that the given renaming is injective, renames the model accordingly, applies the product construction and checks that the certificate is admissible.

If the process is successful, we can conclude by Corollary 1 that no "bad" state $(l, s)$ (i.e. with $F\,l$) is reachable symbolically. We will argue that this really implies that the model is safe in the concrete case of timed automata in section 2.3.

We now lay out how a verified certificate checker that implements said strategy for an abstract transition system can be constructed in Isabelle/HOL. Listing 1.1 displays the definition of the core of the checker that checks whether the certificate is closed in the sense defined above. The program is defined in the non-determinism monad of the Imperative Refinement Framework (IRF) [20]. Some parts, such as checking set membership or converting a (finite) set to a list are still left abstract. A non-deterministic specification $\mathsf{SPEC}\,Q$ returns some value $v$ with $Q\,v$.

The body of the program (lines 2-16) iterates over all discrete states in the certificate $L$ and checks that all corresponding symbolic states are covered. Line 3 retrieves the symbolic states that correspond to discrete state $l$ and in line 4 their symbolic successor states are computed. The result ($\mathit{next}$) is a list of pairs of a discrete state and the set of its corresponding symbolic states. The loop ranging from lines 5 to 15 iterates over this list to ensure that all the successor states are covered. Given a discrete state $l'$ and a set of symbolic states $S'$, line 6 first converts it into a list $xs$ that can be iterated over. This turns into a vacuous operation when the algorithm is refined to an executable version where sets are implemented as lists. Line 8 checks that $l'$ is also part of the certificate. Then, in line 9 the set of corresponding symbolic states is retrieved and converted to a list $ys$. Finally, lines 10-12 ensure that all states in $xs$ are subsumed by some state in $ys$.

To prove soundness of *check*, we mainly need correctness theorems for the monadic combinators *monadic_list_all* and *monadic_list_ex*. Given a list $xs$ and a monadic implementation $Q_i$ of a predicate $Q$, they check whether all states (at least one state) in $xs$ satisfy (satisfies) $Q$. This is the correctness theorem for *monadic_list_all*, for instance:

$$\begin{aligned} & (\forall x.\, Q_i\, x \leq \mathsf{SPEC}\,(\lambda r.\, r \longleftrightarrow Q\, x)) \\ \Longrightarrow \quad & \mathit{monadic\_list\_all}\, xs\, Q_i \leq \mathsf{SPEC}\,(\lambda r.\, r \longleftrightarrow \mathit{list\_all}\, xs\, Q) \end{aligned}$$

where $\mathit{list\_all}\, xs\, Q$ holds if and only if $Q$ holds for all elements in $xs$. After setting up the IRF's verification condition generator with this rule and the corresponding rule for *monadic_list_ex*, it is easy to prove that *check* is sound:

$$\mathit{check}\,(L, M) \leq \mathsf{SPEC}\,(\lambda r.\, r \Longrightarrow \mathit{closed}\,(L, M))$$

where the property $\mathit{closed}\,(L, M)$ corresponds to condition $(*)$ from above.

We then use standard refinement techniques to obtain an algorithm $\mathit{check}_i$ that refines *check*, replacing sets by lists. However, the algorithm is still specified in the non-determinism monad and therefore not executable. We use a simple technique to make it executable. Consider the following theorem for *monadic_list_all*:

$$\mathit{monadic\_list\_all}\, xs\,(\lambda x.\, \mathsf{return}\,(P\, x)) = \mathsf{return}\,(\mathit{list\_all}\, xs\, P)\,.$$

It allows us to replace the non-deterministic combinator *monadic_list_all* by the deterministic *list_all*, pushing return to the outside. By exhaustively applying a set of such rewrite rules we obtain an alternative definition of $\mathit{check}_i$ where return appears only on the outermost level, and the inner term is deterministic and thus executable. Using these techniques, we obtain a simple certificate checker that is executable, provided that we can implement the elementary model checking primitives such as the subsumption check or computing the list of successors of a state.

#### 2.3 Transferring the Correctness Theorem

For timed automata, the abstract transition system studied above is the zone graph $\rightarrow_{ZG(A)}$ of a given (single) automaton $A$. One can show that it simulates $\rightarrow_A$ (completeness of $\rightarrow_{ZG(A)}$):

$$(l, u) \rightarrow_A (l', u') \wedge u \in Z \Longrightarrow (\exists Z'.\, (l, Z) \rightarrow_{ZG(A)} (l', Z') \wedge u' \in Z')\,.$$

This simulation property is sufficient to establish that if there is no reachable state $(l, Z)$ in $\rightarrow_{ZG(A)}$ with $F\, l$, then no final state $(l, u)$ is reachable in $\rightarrow_A$:

$$\begin{aligned} & (\nexists l, Z.\, (l_0, Z_0) \rightarrow^*_{ZG(A)} (l, Z) \wedge F\, l) \wedge u_0 \in Z_0 \\ \Longrightarrow \quad & (\nexists l, u.\, (l_0, u_0) \rightarrow^*_A (l, u) \wedge F\, l) \end{aligned}$$

In the formalization, these proofs rely on instantiating a general theory of simulations in transition systems that is derived from the theory of Wimmer and Lammich [31]. From Corollary 1 we get that there is no reachable final state in $\rightarrow_{ZG(A)}$ if the certificate check is passed. Finally, by correctness of the renaming process and the product construction, we can conclude that there is no final reachable state in the input model if there is no final reachable state in $\rightarrow_A$.

#### 2.4 Implementing a Concrete Checker

All the elementary model checking primitives we need for certification have already been implemented [31]. The abstract implementation presented above assumes that the model checking primitives are implemented in a purely functional manner (as they are just regular HOL functions). The existing (verified) model checker [31], however, is an imperative implementation in the Imperative HOL framework. Imperative HOL [7] is a framework for specifying and reasoning about imperative programs in Isabelle/HOL. It provides a *heap monad* in which one can use—analogously to the ML family of programming languages—imperative references and arrays to express imperative programs. Usually, once we have used an imperative implementation anywhere, the whole program would need to be stated in the heap monad. However, we can employ a technique similar to the one that is used for Haskell's *ST* monad [21] to erase the heap monad in a safe way under certain circumstances.

More precisely, if it can be deduced from the type of an imperative computation that no information about references or arrays on the heap can be leaked to the outside of the computation in its result, then the heap monad can be erased for this computation, yielding a pure computation. In the certifier, this is primarily used for computing the symbolic successor of a zone $Z$ for a certain transition. To that end, an immutable representation of the DBM $M$ corresponding to $Z$ is copied to the a newly allocated imperative array, then the imperative pipeline of computations to compute the successor $M'$ is applied to $M$, and finally $M'$ is copied back to an immutable array. Taken together, this whole computation does not contain the type of an array or reference in its result type, and thus can safely be turned into a pure computation. As a consequence, we are able to reuse the existing verified model checking primitives, while being able to state the certificate checking algorithm purely functionally.

In the concrete checker, the mapping $M$ is implemented using a verified functional hash table implementation based on so-called *diff arrays* [19]. This data structure provides a purely functional interface to an underlying imperative array. When a diff array is updated, it performs the update on the imperative array, and stores a difference that can be used to re-compute the old state of the array. Reading from the most recent version of a diff array is fast as the value can directly be read from the underlying imperative array. If an old version is accessed, the whole array has to be copied to recompute the old version. This gives diff arrays good performance characteristics, as long as they are mostly used linearly. This is the case in our application as the hash table is filled in an initial phase, after which the hash table is used in a read-only manner.

#### 2.5 Parallel Execution

The attentive reader may wonder why we care about a purely functional implementation of the certificate checker at all. Indeed, we could use existing techniques [31] to obtain an imperative implementation of the certificate checker in the heap monad. However, in this setting it would be hard to justify the soundness of executing parts of the checker in parallel. In the purely functional setting, this is much simpler. Our approach to parallel execution is minimalist: we only provide means to execute the *map* combinator on lists in parallel. This is achieved by another custom code translation that is part of the trusted code base. The parallel implementation of *map* uses a task queue that will contain the individual computations that need to be run for each element of *xs*, and uses a fixed number of threads to work through this list and assemble the final result.

We exploit this *map* implementation to work through the list of discrete states $L$ in parallel, using the equivalence:

$$list\_all\, Q\, xs = list\_all\, id\, (map\, Q\, xs)\,.$$

In doing so, we lose the ability to stop execution early once a list element does not satisify $Q$. For the certificate checker, however, we assume that usually the certificate is correct, meaning that we have to go through the whole list anyway. We only parallelize the outermost loop of $check_i$ because this should yield reasonably-sized work portions, given that the size of $L$ will typically at least be in the hundreds.

### 3 Scaling Performance

In this section we discuss two techniques to improve the performance of the certificate checker without increasing the verification effort significantly.

#### 3.1 Monomorphization

Isabelle/HOL supports polymorphism and type classes, which are valuable features for sizeable formalization efforts. Large parts of our formalization also make use of these features, e.g., most of the timed automata semantics are formalized for a general time domain, and operations on DBMs are applicable on DBMs whose entries are formed from more general algebraic structures than the ring of integers. While this yields an abstract and general formalized theory, it can get in our way when trying to obtain efficient code.

When generating SML code from HOL, Isabelle uses a so-called dictionary construction to compile out type classes, which are not supported by SML. This means that most functions carry a large number of additional parameters, which are used to look up elementary operations, such as addition of two numbers. These additional lookup operations degrade performance. One solution is to ensure that all relevant constants that are exported to SML are monomorphic (i.e. specialized to the integer type), eliminating the need for the dictionary construction in most places. Thus, we apply a semi-automated procedure to achieve this monomorphization.

#### 3.2 Integer Representation

Types such as *int* or *nat* are unbounded in Isabelle/HOL meaning they are implemented with the help of big integers in the target languages. To improve performance, we want to use machine integers instead, and instruct Isabelle/HOL's code generator to do that. This is still sound: SML's standard integer operations throw an exception if an overflow occurs instead of silently wrapping around. The code generator can only achieve partial correctness anyway: if program execution does not fail, then its result is consistent with the evaluated HOL term.

#### 3.3 Refined Code Equations

The last type of optimizations we use can be considered to belong to the category of micro-optimizations. These are improved code generator translations for elementary operations and combinators. We employ such improved translations to use native implementation language primitives to convert from mutable to immutable arrays and back. The other such optimizations we use, is to directly use integer values as counters in imperative loops instead of a natural number representation that would box the integers in a data constructor. In the same way, we use integers directly for array indexing.

### 4 Certificate Compression

In this section, we present two techniques to compress the unreachability certificate. By compression we mean reducing the number of zones that are present in the certificate for each discrete state, using the unverified model checker. The first technique relies on subsumption. As explained above, it is possible that the model checker adds a zone $Z$ to the set of explored states and later another zone $Z'$ with $Z \subseteq Z'$ (i.e. $Z'$ subsumes $Z$). Thus the first technique simply filters the set w.r.t. $\subseteq$ in the end.

The second technique relies on the following idea: we replace one or more zones by their union, and check that the state space is still closed. This means that we have to check that all the successors of the larger zone are still covered by the current set of states. In that case, we can discard the old zones, and replace them by their union. As the union of two zones is not necessarily convex and thus cannot be represented as a DBM, we do not compute a precise union of zones but their convex hull. This operation is rather cheap as it amounts to taking the pointwise maximum of DBM entries. After computing the convex hull of a number of zones (in canonical form), we only need to apply the expensive operation to restore a canonical form once.

The latter technique yields a whole family of compression algorithms by iterating one of the following operations for each discrete state until a fixed-point is reached:

a) the convex hull of all zones is computed;
b) the convex hull of the first two zones is computed; c) the convex hull of the first two zones that can successfully be joined is put to the front of the list;
d) same as c) but considering only discrete states for which compression was successful in the last round;
e) same as d) but iterating the operation until saturation.

The next section contains an experimental evaluation of these techniques.

Note that similar techniques for reducing the search-space could also be applied already during model checking. By doing so, the number of states explored and the runtime of model checking could be reduced. This, however, comes at the risk of producing spurious model checking results (i.e. a final state might be deemed reachable, although there is no corresponding reachable state in the timed automaton).

### 5 Experimental Evaluation

We evaluate the checker on a set of benchmarks that is derived from Uppaal's standard benchmark suite [22]. Additionally, to cover the advanced modeling features of committed locations and broadcast channels, we use a set of benchmarks that is derived from the pacemaker models of Jiang et al. [17] and a modified version of the FDDI benchmark with broadcast channels. A prototype SML implementation of a timed automata model checker (Mlunta) is used to compute the certificates. We use reachability properties of the form $\mathbf{E}\Diamond$ *false* to enforce that the model checker explores the complete state space. The results are given in Table 1. The problem size is specified as the number of automata in the network. We report the total runtime (wall time) of:

1. the tandem consisting of Mlunta (using the first compression technique) and the (verified) certificate checker, both compiled with MLton;
2. the individual runtime of the (verified) certificate checker for a varying number of threads for parallel computation, compiled with Poly/ML as it is the only SML compiler that supports multi-threading;
3. the runtime of Uppaal configured for depth-first search (like Mlunta);
4. the runtime of an unverified SML implementation of the certificate checker based on Mlunta (compiled with MLton);
5. and the runtime of the fully verified model checker Munta [31] extended with the improvements from sections 2 and 4 and compiled with MLton.

As can be seen from the results, the tandem is still one order of magnitude slower than Uppaal, but certificate checking in isolation is also up to one order of magnitude faster than the previous verified model checker [31]. Note that Mlunta explores significantly more states than Uppaal and Munta for "Pacemaker". Multi-core scale beyond two threads is relatively unsatisfactory, however. In micro-benchmarks, we have identified that the problem appears to be with memory allocation on the heap, even if no data is shared among threads (in our case, only the certificate is shared but successors are computed locally). There does not seem to be an obvious way to improve on this situation for SML implementations. Finally, one can see that the verified certifier is not drastically slower than the unverified implementation based on Mlunta, indicating that the verified certifier is not missing any obvious significant optimizations.

| | | | | | | Certifier for #threads | | | | |
|---|---|---|---|---|---|---|---|---|---|---|
| Model | Size | Uppaal | Tandem | Munta | Unverif. | 1-MLton | 1 | 2 | 3 | 4 |
| FDDI | 8 | 0.33 | 0.79 | 1.01 | 0.14 | 0.21 | 0.99 | 0.64 | 0.57 | 0.53 |
| | 10 | 5.93 | 1.77 | 2.50 | 0.40 | 0.45 | 2.13 | 1.36 | 1.26 | 1.20 |
| | 12 | 92.66 | 3.93 | 5.42 | 0.90 | 1.20 | 3.41 | 2.33 | 2.25 | 2.18 |
| | 14 | 1874.28 | 7.28 | 10.73 | 1.86 | 2.22 | 5.36 | 3.94 | 3.90 | 3.88 |
| | 16 | *** | 12.80 | 19.51 | 3.47 | 3.72 | 11.09 | 6.49 | 6.50 | 6.51 |
| FDDI broad | 8 | 0.34 | 0.28 | 1.07 | 0.10 | 0.08 | 0.26 | 0.19 | 0.17 | 0.16 |
| Fischer | 5 | 0.24 | 2.78 | 6.33 | 0.72 | 0.53 | 1.76 | 1.07 | 0.98 | 0.91 |
| | 6 | 34.74 | 143.72 | 377.70 | 40.99 | 26.58 | 40.60 | 25.47 | 24.16 | 23.67 |
| CSMA | 5 | 0.04 | 0.94 | 4.42 | 0.31 | 0.28 | 1.44 | 0.87 | 0.80 | 0.76 |
| | 6 | 1.53 | 13.48 | 65.16 | 5.24 | 4.18 | 12.16 | 8.04 | 7.87 | 7.76 |
| | Mode | | | | | | | | | |
| Pacemaker | 1 | 0.02 | 0.16 | 0.37 | 0.03 | 0.03 | 0.25 | 0.16 | 0.14 | 0.13 |
| | 2 | 0.02 | 0.75 | 3.20 | 0.17 | 0.26 | 1.23 | 0.75 | 0.68 | 0.65 |
| | 3 | 0.03 | 1.39 | 4.23 | 0.34 | 0.46 | 2.53 | 1.55 | 1.40 | 1.31 |
| | 4 | 0.02 | 11.80 | 0.70 | 3.24 | 3.71 | 12.13 | 8.38 | 6.69 | 6.66 |
| | 5 | 0.02 | 30.84 | 0.86 | 9.13 | 10.07 | 26.60 | 18.58 | 18.15 | 17.95 |

Table 1: Benchmarks results on a machine with 16 GB RAM and an Intel(R) Core(TM) i7-4610M CPU at 3.00GHz with two cores and two threads per core. The column labeled "Tandem" gives the runtime for a combination of the unverified SML tool and the verified certificate checker. The next column gives the runtime of the unverified SML certifier, followed by the runtimes of the verified checker for a varying number of threads. All times are given in seconds.

Table 2 gives the results of evaluating the different compression algorithms on the same set of benchmarks. The second variant is always applied to the compression result of the first variant to avoid trivial computations of the convex hull. Variant 2c) (the most expensive one) can produce drastically smaller certificates than any other variant, and its minimum compression factor is an order of a magnitude higher than for any other variant. Nevertheless, only variants 1 and 2a) appear to be useful in practice, as they are relatively cheap to compute. The other variants could prove useful if the certificates were produced by a significantly more efficient model checker, such as Uppaal or TChecker [15]. On a final note, we have constructed a more than 95% smaller but valid certificate for the Fischer benchmark, suggesting that there is room for improvement on the compression algorithms.

| | | Variant | | | | | |
|---|---|---|---|---|---|---|---|
| Model | Size | 1 | 2a | 2b | 2c | 2d | 2e |
| FDDI | 8 | 0.21 | 0.21 | 1.72 | 69.53 | 3.65 | 3.43 |
| FDDI broadcast | 8 | 0.00 | 48.94 | 48.94 | 48.94 | 1.06 | 1.06 |
| Fischer | 5 | 22.03 | 22.03 | 22.72 | 43.06 | 30.40 | 30.40 |
| CSMA/CD | 5 | 26.06 | 41.54 | 43.84 | 81.16 | 58.94 | 47.54 |
| | 6 | 24.86 | 41.91 | 44.02 | 88.35 | 63.24 | 47.02 |
| | Mode | | | | | | |
| Pacemaker | 1 | 16.07 | 25.00 | 30.80 | 58.04 | 29.02 | 29.02 |
| | 2 | 24.00 | 26.38 | 30.37 | 58.68 | 35.87 | 35.22 |
| | 3 | 12.96 | 17.62 | 19.23 | 46.92 | 25.30 | 25.01 |
| | 4 | 13.82 | 20.02 | 23.60 | 41.48 | 26.16 | 24.71 |
| | 5 | 17.14 | 22.48 | 25.46 | 39.69 | 28.18 | 26.88 |
| Average | | 15.71 | 26.61 | 29.07 | 57.58 | 30.18 | 27.03 |

Table 2: Certificate compression factors (given in %).

### 6 Conclusion and Future Work

We have presented a verified certifier of unreachability certificates for a timed automata. The certificates are ought to be produced by an unverified model checker. Experimentation shows that verified certificate checking in isolation is up to an order of magnitude faster than what was previously possible with a verified model checker [31]. The performance of a tandem of an unverified model checker and the verified certifier could be improved by replacing the certificate-producing part with a highly optimized tool, possibly opening room to use some of the more powerful certificate compression techniques we suggested above. As we pointed out above, there appears to be further room for improvement on the certificate compression algorithms as well.

Moreover, more sophisticated tools also employ more powerful abstraction techniques, for which our proposed certification technique is still suitable—to a large extent without requiring additional verification effort. An exception is the implicit abstraction technique studied by Herbreteau et al. [14] as it does not compute abstractions of zones explicitly but rather checks subsumptions of the form $Z \subseteq \alpha(Z')$ implicitly, meaning that one would have to prove correctness of the subsumption check to validate certificates produced by such a model checking process.

Finally, we intend to extend this work to certification of emptiness of timed Büchi automata in the future, using the idea of *subsumption graphs* [13] and relying on an unverified model checker implementation for timed Büchi automata to produce the certificates [13,18].

### Data Availability Statement

The datasets generated and/or analyzed during the current study are available in the Zenodo repository [32]: https://doi.org/10.5281/zenodo.3679245. The artifact has been tested on the TACAS artifact evaluation VM [12].
